# Applied ecoimmunology: using immunological tools to improve conservation efforts in a changing world

**DOI:** 10.1093/conphys/coab074

**Published:** 2021-09-07

**Authors:** Michel E B Ohmer, David Costantini, Gábor Á Czirják, Cynthia J Downs, Laura V Ferguson, Andy Flies, Craig E Franklin, Ahab N Kayigwe, Sarah Knutie, Corinne L Richards-Zawacki, Rebecca L Cramp

**Affiliations:** 1 Living Earth Collaborative, Washington University in St. Louis, MO 63130, USA; 2 Unité Physiologie Moléculaire et Adaptation (PhyMA), Muséum National d’Histoire Naturelle, CNRS, 57 Rue Cuvier, CP32, 75005, Paris, France; 3 Department of Wildlife Diseases, Leibniz Institute for Zoo and Wildlife Research, 10315 Berlin, Germany; 4 Department of Environmental Biology, SUNY College of Environmental Science and Forestry, Syracuse, NY 13210, USA; 5 Department of Psychology and Neuroscience, Dalhousie University, Halifax, NS B3H 4R2, Canada; 6 Menzies Institute for Medical Research, University of Tasmania, Tasmania 7001, Australia; 7 School of Biological Sciences, The University of Queensland, Queensland 4072, Australia; 8 Department of Ecology and Evolutionary Biology, University of Connecticut, Storrs, CT 06268, USA; 9 Institute for Systems Genomics, University of Connecticut, Storrs, CT 06268, USA; 10 Department of Biological Sciences, University of Pittsburgh, Pittsburgh, PA 15260, USA

## Abstract

Ecoimmunology is a rapidly developing field that explores how the environment shapes immune function, which in turn influences host–parasite relationships and disease outcomes. Host immune defence is a key fitness determinant because it underlies the capacity of animals to resist or tolerate potential infections. Importantly, immune function can be suppressed, depressed, reconfigured or stimulated by exposure to rapidly changing environmental drivers like temperature, pollutants and food availability. Thus, hosts may experience trade-offs resulting from altered investment in immune function under environmental stressors. As such, approaches in ecoimmunology can provide powerful tools to assist in the conservation of wildlife. Here, we provide case studies that explore the diverse ways that ecoimmunology can inform and advance conservation efforts, from understanding how Galapagos finches will fare with introduced parasites, to using methods from human oncology to design vaccines against a transmissible cancer in Tasmanian devils. In addition, we discuss the future of ecoimmunology and present 10 questions that can help guide this emerging field to better inform conservation decisions and biodiversity protection. From better linking changes in immune function to disease outcomes under different environmental conditions, to understanding how individual variation contributes to disease dynamics in wild populations, there is immense potential for ecoimmunology to inform the conservation of imperilled hosts in the face of new and re-emerging pathogens, in addition to improving the detection and management of emerging potential zoonoses.

## Teaser

Immunological tools have enormous potential to help us understand and manage wildlife diseases in a rapidly changing world. Here, we present a series of case studies in which immunological tools have been used to inform conservation actions. Additionally, we identify opportunities to improve the utility of ecoimmunological tools to conservation practitioners.

## Introduction: the role of ecoimmunology in conservation

Ecoimmunology is a rapidly expanding field that aims to investigate the causes and consequences of variation in immunity within an ecological and evolutionary framework (Downs and Stewart, 2014; Schoenle *et al.*, 2018). By bridging the gap between disease ecology and traditional ecophysiology, ecoimmunology melds the measurement of immunological markers with disease dynamics at multiple scales and over space and time to understand how environmental conditions influence susceptibility to and prevalence of disease (Brock *et al.*, 2014). Broadly, the immune system serves as an organism-level process that monitors environmental changes and responds to threats to physiological homeostasis, such as parasites, pathogens and cancer. As the climate changes, the emergence and spread of new and existing parasites and pathogens poses a significant risk not only to human life and animal health, but also to the conservation of wildlife (Altizer *et al.*, 2013; Cohen *et al.*, 2019a; Ryan *et al.*, 2019). Through the measurement and understanding of immunological defences and processes, we can better predict the impacts of emerging diseases and zoonoses.

As ecoimmunological tools continue to be validated and refined for use in wildlife, there is considerable application for conservation. Going beyond simply monitoring pathogens in wildlife, we can use ecoimmunology to manage emerging pathogens, monitor and predict disease susceptibility and understand or better predict remediation success (Downs and Stewart, 2014; Cramp and Franklin, 2018). For example, traditional ecoimmunological tests, such as swelling response to an antigen or measurements of antibodies, can be paired with experimental manipulations and measurements of parasite load both in the field and the laboratory to understand when immunological resistance is effective against parasites (see Parasite invasions and immunity in Galapagos finches). By measuring immunological and physiological markers in wildlife, we can determine how they are associated with disease and/or different fitness outcomes or how the individual physiological status can constrain the immune response (see Biomarkers of health, stress, and disease status in frigatebirds) and respond with the best conservation approaches to improve wildlife health. Finally, ecoimmunology can meld wildlife conservation approaches with advances in human medicine to develop and effectively administer vaccines for species of conservation concern (see Using immunology to understand transmissible cancers in Tasmanian devils).

An ecoimmunological perspective will become even more important as we disentangle and respond to the effects of ever-increasing anthropogenic stressors on host–pathogen interactions and disease emergence. Immune function is often modulated by temperature and seasonality (Martin *et al.*, 2008), both of which are shifting with climate change. Increasingly high and variable global temperatures are modifying seasonal patterns of behaviour and physiology, which can create mismatches between the environment and immune function capacity, or carry-over effects of shifting early environments on immune function development (see Impacts of a changing climate on the development of amphibian immune defences). Furthermore, determining the mechanistic link between the microbiome, which has been shown to relate to many aspects of host health (Levy *et al.*, 2017; Trevelline *et al.*, 2019), and immune function will help elucidate the causes and consequences of changing environmental conditions on disease dynamics (see Lessons from cricket symbionts and pathogens: connecting microbes to the role of ecoimmunology in conservation biology). Additionally, understanding the role of nutritional condition and feeding supplementation on parasite infections can improve and expand remediation responses (see Nutritional status and immunity), particularly as the spread of urban environments can increase non-natural food resources and lead to behavioural challenges for wildlife. Finally, uncovering unique aspects of the bat immune system can help us understand what makes these animals susceptible to a devastating fungal disease, white nose syndrome, yet simultaneously makes them important vectors of viruses that can infect humans (see Ecoimmunology and disease ecology in bats).

In this perspective piece, we provide case studies across various taxonomic groups that demonstrate the depth and breadth of current and future work at the intersection of ecoimmunology and applied conservation biology in a changing world. Then, we discuss what we feel are the most important ‘big questions’ facing the field of ecoimmunology, from how to better integrate the study of immunity and other biological and physiological functions, to understanding how individual differences in immunity scale up to population-level effects. For each of these questions, we discuss the future of the field and the steps we can take to tackle them.

## Applied ecoimmunology in conservation: case studies

### Parasite invasions and immunity in Galapagos finches

Birds in the Galapagos Islands have faced many novel challenges, including introduced parasites and pathogens, during the Anthropocene (Wikelski *et al.*, 2004). For example, Darwin’s finches are currently dealing with an onslaught of invasive parasites, such as avian poxvirus (Kleindorfer and Dudaniec, 2006; Parker *et al.*, 2011) and avian vampire flies (*Philornis downsi*) (Kleindorfer and Dudaniec, 2016; McNew and Clayton, 2018). These parasites can cause significant mortality (up to 100%) in Darwin’s finches, at least in part because finches are naïve to these novel parasites (Koop *et al.*, 2013; O’Connor *et al.*, 2014).

Exploring the role of ecoimmunology in these host-invasive parasite interactions has improved our understanding of how Darwin’s finches are able to deal with new challenges. For example, avian poxvirus prevalence in small ground finches (*Geospiza fuliginosa*) has been shown to increase with island size, which coincides with an increase in the antibody response (to an immune-stimulatory protein, keyhole limpet haemocyanin) and a decrease in the cell-mediated immune response [phytohaemagglutinin (PHA) skin test; Lindström *et al.*, 2004]. Darwin’s finches also produce parasite-binding IgY antibodies to the invasive vampire flies (Huber *et al.*, 2010). Adult flies are non-parasitic but they lay their eggs in the nests of the birds (McNew and Clayton, 2018). Once the fly eggs hatch, the larval instars feed on the blood and other fluids of the nestling and adult brooding female birds. Small and medium ground finch nestlings generally do not produce a detectable parasite-binding IgY antibody response to the parasite (Koop *et al.*, 2013; Knutie *et al.*, 2016). However, when researchers experimentally manipulated parasite presence, they found that parasitized female finches produced a higher antibody response, compared to non-parasitized females (Koop *et al.*, 2013). Furthermore, higher antibody levels in parasitized females correlated negatively with parasite abundance (Koop et al., 2013). These results suggest that this acquired immune response is not developed until juvenile finches leave their nests and that effective parasite resistance could depend significantly on the brooding mother.

These studies show that Darwin’s finches produce measurable innate and adaptive immune responses to invasive parasites. Darwin’s finches host other parasitic taxa, such as lice and mites, with which they have longer-standing relationships (reviewed in Bulgarella *et al.*, 2017). Therefore, finches could have evolved immune defences against their native parasites, which also cross-react with the novel parasites. Although finches produce many types of immune responses to their parasites, studies have yet to experimentally demonstrate whether and/or when immunological resistance is effective against novel invasive parasites.

Darwin’s finches have also had to deal with habitat change resulting from the rising human resident and tourist population in the Galapagos (Salinas-de-León *et al.*, 2020). Urban finches can experience an increase in fitness compared to non-urban finches (Harvey *et al.*, 2021), which might be related to increased food availability (De León *et al.*, 2019) or changes in behaviour (Gotanda, 2020) or the gut microbiota (Knutie *et al.*, 2019). This habitat change could improve finch immunity to parasites by providing the necessary energetic resources to invest in an immune response, as found in other bird-parasite systems (Knutie, 2020). Preliminary evidence suggests that urban finches have higher nesting success than non-urban finches, which is likely related to their improved resistance to avian vampire flies (Harvey *et al.*, 2021). However, human-altered habitats can have varying effects on finch-parasite interactions. For example, Darwin’s finches in agricultural areas can experience different pox infection dynamics across years, compared to finches in non-agricultural areas, which is likely related to changes in the finches’ innate immune function (Zylberberg *et al.*, 2013). Although the effect of human activity on Darwin’s finches has garnered more attention recently (De León *et al.*, 2019; Knutie *et al.*, 2019; Gotanda, 2020), more studies are needed to understand how humans are affecting immunological resistance in finches against invasive parasites. Identifying immunologically resistant and non-resistant populations of finches could help determine where to focus management efforts, as well as understand what environmental factors promote the evolution of resistance in particular populations.

### Biomarkers of health, stress and disease status in frigatebirds

Viruses are a global conservation concern for avian populations because they are responsible for a variety of pathological effects in birds (Thomas *et al.*, 2007). The case of magnificent frigatebirds (*Fregata magnificens*) breeding in French Guiana represents one recent example of a conservation challenge due to disease. The French Guiana population of magnificent frigatebirds is one of the most important in South-America because of its size and its position between the populations of the Caribbean and Brazil. Outbreaks of a viral disease, associated with an emergent alphaherpesvirus, have occurred annually since 2005 and cause high mortality rates in chicks (De Thoisy *et al.*, 2009; Sebastiano *et al.*, 2017a; Sebastiano *et al.*, 2017b). Bacterial cultures and microscopic evaluation of skin samples and viral screening excluded the presence of ectoparasites, avian poxvirus and avian influenza (De Thoisy *et al.*, 2009). However, laboratory screening enabled the detection of the DNA of a novel alphaherpesvirus in body crusts (De Thoisy *et al.*, 2009) and a strong replication of the virus in beak or cloacal swabs collected from chicks with clinical signs of the disease (nodular proliferative skin lesions). These results show that herpesvirus replication is involved in the appearance of clinical signs in chicks (Sebastiano *et al.*, 2017b).

Sebastiano *et al*. (2017a, 2017b, 2018) found that visible clinical signs of the disease are significantly associated with higher concentrations of a blood-based marker of inflammation (the acute-phase protein haptoglobin) and of a blood-based marker of lipid oxidative damage as compared to healthy chicks. Both markers were also associated with the short-term survival probability of chicks: birds with higher haptoglobin or oxidative damage were those with the lowest probabilities of survival (Sebastiano *et al*. 2017a, 2017b). In addition, compared to chicks without clinical signs, those showing severe clinical signs had higher blood concentrations of both reduced and oxidized glutathione (intracellular antioxidant) and higher haemagglutination and haemolysis scores (indicating higher levels of natural antibodies) (Sebastiano *et al.*, 2018). Supplementation of resveratrol, which is a polyphenol with antioxidant and antiviral properties, increased the concentration of haptoglobin in plasma at an earlier phase of the disease, increased circulating antioxidant defences in healthy chicks and reduced generation of lipid oxidative damage in sick chicks (Sebastiano *et al*., 2018). This suggests that resveratrol is rapidly metabolized in sick chicks to control levels of lipid peroxidation (explaining the lack of increase of circulating antioxidant defences) (Sebastiano *et al*., 2018). Furthermore, resveratrol sustained the production of nitric oxide and had negligible to no influence on haemolysis and haemagglutination scores, baseline corticosterone and activity of antioxidant enzymes (Sebastiano *et al.*, 2018). Overall, these studies showed that chicks with visible clinical signs of the disease have a pronounced alteration in particular components of their immune and oxidative statuses in comparison with birds without clinical signs. Moreover, these studies showed that regulation of certain immunological mechanisms (including inflammation) and of oxidative status are strictly intertwined and contribute to elucidating the mechanisms underlying the host-pathogen interaction. In conclusion, these studies suggest that relying on physiological markers that do not require complex laboratory analyses would enable (i) continuous monitoring of the health status of a target population and (ii) the rapid assessment of the efficiency of any intervention (e.g. pharmacological treatment), because physiological markers respond faster than other metrics, such as visible clinical signs.

### Using immunology to understand transmissible cancers in Tasmanian devils

Transmissible cancers provide a unique opportunity to probe immune evasion mechanisms used by cancer cells, host-pathogen coevolution and immunological tolerance to genetically mis-matched tissues in a natural ecological setting. Tasmanian devils (*Sarcophilus harrisii*) are affected by two independent transmissible cancers known as devil facial tumours (Pearse and Swift, 2006; Pye *et al.*, 2016). The first devil facial tumour (DFT1) was reported in 1996 and has now spread across most of the devil’s geographic range, reducing the devil population by 77% (Lazenby *et al.*, 2018). The second devil facial tumour (DFT2) was discovered in 2014 and thus far exists only in southern Tasmania (Pye *et al.*, 2016). Using an evolutionary framework and drawing on techniques from studies of human oncology, there has been a considerable effort not only to understand the drivers of clinical disease in wild devils, but possible methods to control these cancers via vaccination and/or immunotherapy.

Major histocompatibility complex class I (MHC-I) is a major target of allograft rejection in humans and reduced MHC-I expression is an immune evasion mechanism employed by many human cancers (Yoshihama *et al.*, 2016). Thus, low genetic diversity in MHC-I and MHC-II alleles was initially proposed as means of DFT1 cells avoiding allograft immune responses; many devils do not have the expected set of six classical MHC-I alleles and a subset of devils lack functional copies of the Saha-UA alleles (Cheng *et al.,* 2012). However, skin graft experiments demonstrated that devils do reject allograft skin but not autograft skin (Kreiss *et al.,* 2011). Likewise, mixed lymphocyte reactions yielded the strongest responses when blood was obtained from geographically separated devil subpopulations as opposed to weak responses from devils in the same subpopulation that are more likely to have similar MHC alleles (Kreiss *et al.,* 2011). Functional studies at the protein level have shown that DFT1 cells do not constitutively express MHC-I on the cell surface (Siddle *et al.*, 2013). Interestingly, this epigenetic downregulation of MHC-I is reversible upon treatment with interferon-gamma. DFT1 cells also have a hemizygous deletion of the *B2M* gene that is needed for MHC-I expression, which is hypothesized to be associated with reduced MHC-I expression on DFT1 cells (Stammnitz *et al.*, 2018). DFT2 cells discovered soon after the emergence of DFT1 do express MHC-I, but the alleles that are most highly expressed *in vivo* appear to be alleles that match host classical MHC-I or are less polymorphic non-classical MHC-I genes (Caldwell *et al.*, 2018).

Experimental vaccines and immunotherapies based on upregulation of MHC-I have confirmed that anti-DFT1 immunity can be induced (Tovar *et al.*, 2017), but to date they have not been able to prevent DFT1 infections. Natural DFT1 regressions have also been observed in wild devils (Pye *et al.*, 2016; Margres *et al.*, 2018). CRISPR/Cas9 was used to completely knockout MHC-I from DFT1 cells, and this cell line was used to show that MHC-I proteins are a major serum antibody target in devils that had immunotherapy-induced or natural DFT1 regressions (Pye *et al.*, 2016; Margres *et al.*, 2018; Ong *et al.*, 2021). Altogether these results suggest that low MHC-I diversity and reduced MHC-I expression are important for transmissible cancer cells, but additional mechanisms are likely employed to evade immune defences.

Immunotherapies targeting immune checkpoint proteins have become a pillar of human oncology, and many of the key immune checkpoint interactions and expression patterns appear to be conserved in devils (Flies *et al.*, 2016; Flies *et al.*, 2017; Flies *et al.*, 2020a). Additionally, drugs for human cancers that target receptor tyrosine kinases have yielded promising *in vitro* results for DFT1 and DFT2 (Kosack *et al.*, 2019). However, these treatments have limited potential to impact devil conservation due to the need to repeatedly trap and inject devils. Vaccines delivered in food baits that can be distributed across the landscape have played a key role in rabies control for more than 50 years (Mueller *et al.*, 2015). This approach is being explored for several other wildlife diseases, including DFT1 and DFT2 (Chambers *et al.*, 2017; Rocke *et al.*, 2017; Rocke *et al.*, 2019; Flies *et al.*, 2020b). The ongoing ecological monitoring can inform vaccine distribution strategies if an effective vaccine is developed.

### Impacts of a changing climate on the development of amphibian immune defences

Infectious diseases have ravaged amphibian populations worldwide, and chytridiomycosis has been the most devastating to date (Scheele *et al.*, 2019). The causal pathogens, chytrid fungi *Batrachochytrium dendrobatidis* (*Bd*) and *Batrachochytrium salamandrivorans*, have spread via the food and pet trade worldwide (Berger *et al.*, 1998; Martel *et al.*, 2013; O’Hanlon *et al.*, 2018), and there is growing evidence that environmental change is contributing to the frequency and intensity of chytridiomycosis outbreaks. Environmental temperature is a strong driver of infection dynamics in the field (Sonn *et al.*, 2017; Cohen *et al.*, 2019b; Sonn *et al.*, 2019), and both the fungal pathogen and amphibian immune function are thermally sensitive (Piotrowski *et al.*, 2004; Sonn *et al.*, 2017; Sauer *et al.*, 2018; Robak *et al.*, 2019). Interactions with *Bd* also impact host physiology in ways that suggest synergistic effects between disease and other stressors. For example, infection increases rates of evaporative water loss through the skin and skin sloughing in adult amphibians and lowers the maximum temperatures animals can withstand (Ohmer *et al.*, 2015; Greenspan *et al.*, 2017; Ohmer *et al.*, 2017; Russo *et al.*, 2018), increasing their risk of thermal and hydric stress. Recent work comparing climate anomalies with locations of declines indicates that a changing climate has likely played a role in disease outbreaks and species extinctions (Rohr and Raffel, 2010; Cohen *et al.*, 2019a), highlighting the potential for an increasing impact of disease on amphibians under future climate scenarios.

As ectotherms, amphibians are vulnerable to a changing climate, both in terms of rising/more variable temperatures and shifts in water availability (Raffel *et al.*, 2013; Altman *et al.*, 2016; Raffel et al., 2015). Amphibian larvae develop in unpredictable environments and the stressors they encounter at this life stage can impact the formation of immune defences as adults (Kohli *et al.*, 2019). As air temperatures increase, alterations to the global water cycle are predicted to result in more frequent droughts and reduced hydroperiods (Milly *et al.*, 2005; McMenamin *et al.*, 2008). As ponds dry (Walls *et al.*, 2013), amphibians may respond plastically by shortening their larval period to escape impending desiccation (Richter-Boix *et al.*, 2011; Edge *et al.*, 2016). This can result in trade-offs between larval and post-metamorphic growth, immune function and body condition, which have been shown to reduce fecundity and survival into adulthood (Wilbur, 1987; Semlitsch *et al.*, 1988; Berven, 1990; Kohli *et al.*, 2019).

We have increasing evidence that the developmental environment can have lasting impacts on amphibian immune function, demonstrating the importance of investigating the impacts of environmental change and disease across multiple life stages. In the wood frog (*Rana sylvatica*), Gervasi and Foufopoulos (2008) found that there was an immunological cost to increasing developmental rate in response to pond drying. After metamorphosis, animals reared as larvae under drying regimes had lower cell-mediated immune responses to PHA and reduced leukocyte counts, demonstrating a potential trade-off between accelerating development and immune function (Gervasi and Foufopoulos, 2008). Northern leopard frogs (*Rana pipiens*) reared under pond drying also showed dampened cell-mediated responses to PHA injection (Brannelly *et al.*, 2019). In addition, animals with shorter larval periods had reduced total antibody production and lower bacterial killing ability of whole blood after metamorphosis (Brannelly *et al.*, 2019). Finally, when exposed to *Bd*, frogs that had experienced drying during development exhibited lower survival (Ohmer *et al*., in prep.). Collectively, this work provides evidence of the insidious effects of a changing climate on amphibian susceptibility to pathogens and highlights the importance of considering carry-over effects from sensitive life stages when designing management plans for species at risk of disease-related declines.

### Lessons from symbionts and pathogens in the cold: connecting microbes to the role of eco-immunology in conservation biology

Environmental challenges and changes can directly impact both immune function and the physiology of microbes, and consequently the outcome of their interactions (Ferguson *et al.*, 2018a; Jin Song *et al.*, 2019). Changes in symbiotic microbial communities can thus impact host disease resistance (Knutie *et al.*, 2017a; Knutie, 2018; Knutie, 2020), disease transmission (Weiss and Aksoy, 2011; Hegde *et al.*, 2015), networks of physiological activity connected to immunity (Ferguson *et al.*, 2018b) and the overall ability to thrive in a changing environment (West *et al.*, 2019). Conservation biology would benefit from the ability to predict the outcomes of these host–microbe interactions such that we can foresee and mitigate changes in the dynamics of infection. Further, we may use this understanding to effectively supplement hosts with beneficial microbes (e.g. bioaugmentation) to boost host resilience under challenging environments (Jin Song *et al.*, 2019). Thus, ecoimmunology will be increasingly effective in conservation biology if it integrates an understanding of the impact of environmental change on the microbes that support and challenge their hosts.

To use ecoimmunology and host–microbe interactions to predict host success under changing environments, we need a mechanistic understanding of their connection (Trevelline *et al.*, 2019). For example, overwintering modifies the community of microbes in the gut of *Gryllus veletis* crickets, notably reducing populations of *Pseudomonas* spp. Simultaneously, winter depresses various measures of immunity in both *G. veletis* (Ferguson *et al.*, 2018a) and a variety of other insects (Ferguson and Sinclair, 2020). Because *Pseudomonas* spp. can be opportunistic pathogens, as well as ice nucleators (Olsen and Duman, 1997) it is possible that the host prophylactically modifies the microbiome to reduce the chance of infection or freezing damage during a period of dormancy (Ferguson *et al.*, 2018a). Alternatively, if low temperatures or seasonal changes in the physiology of the gut reduce the chance of opportunistic infections, this could permit the host to down-regulate its investment in immunity over the winter and shuttle resources to other physiology such as cold tolerance (Ferguson *et al.*, 2018a). The potential impact of this concerted seasonal shift in immunity and the microbiome on host success thus depends on how vulnerable this connection will be to environmental change. Changing winter conditions may lead to new shifts in the microbiome that could impact immunosuppressed hosts. Alternatively, shifts in immune activity could impact how the microbiome is mediated seasonally. Thus, it is important to understand if environmental cues trigger hosts to prophylactically modify their microbiomes, if environmentally mediated changes in the microbiome trigger hosts to modify their immune investment, and how tightly these host–microbe interactions will be maintained under shifting environments. We suggest that characterizing plasticity in microbiomes will create opportunities to explore the connection to host health, as well as the efficacy of conservation efforts that include microbial supplementation.

Understanding host resilience and disease dynamics through ecoimmunology will be most useful if we also consider the physiology of the microbes that they encounter—both their symbionts and their pathogens. For example, in the same species of crickets (*G. veletis*), the outcome of infection with the entomopathogenic fungus *Metarhizium brunneum* changes depending on the thermal acclimation and thermal performance of both the host and the pathogen (Ferguson and Sinclair, 2020). Fluctuating temperatures appear to improve immune function in the host; however, cold acclimation in the fungus negates these benefits (Ferguson and Sinclair, 2020). Thus, if we only understand changes in the host immune system, we miss out on how these changes ultimately impact the outcome of infection. Therefore, using ecoimmunology to make predictions about host resilience and emerging diseases will benefit from a physiological understanding of the microbes that interact with the immune system. We suggest that studies aiming to make these predictions should consider that both hosts and pathogens/beneficial symbionts to respond to environmental change.

### Nutritional condition and immunity

The immune system evolved to defend against parasites, and variation in immunological phenotypes can govern population-level dynamics of both infectious disease and host populations (Lochmiller, 1996; Hawley and Altizer, 2011; Martin *et al.*, 2016). Still, what the consequences of individual variation for disease spread may remain an unanswered question in disease ecology (Becker *et al.*, 2019). Host competence—an individual’s ability to perpetuate a parasite (Paull *et al.*, 2012)—is influenced by condition (Beldomenico and Begon, 2010), including nutritional condition, which provides the most direct and sensitive measure of resource limitation for the organism’s functions (Parker *et al.*, 2009; Monteith *et al.*, 2013; Monteith *et al.*, 2014). Understanding how nutritional condition mediates immune defences is critical for understanding the population dynamics of species and associated disease dynamics (Downs and Stewart, 2014).

Superficially, the relationship between nutritional condition and immune defences appears quite simple. Animals in high nutritional condition should invest in immune defences in a manner that optimally balances protection against parasites with immunopathology (Downs *et al.*, 2014; Downs and Stewart, 2014), and in general infection occurrence and intensity is more severe in individuals in poor condition (Beldomenico and Begon, 2010). This pattern often holds when nutritional condition is manipulated through food restriction (e.g. French *et al.*, 2007a; French *et al.*, 2007b), and resource-based reductions of immunity can be ameliorated by supplementing with the restricted resource (Ruiz *et al.*, 2010; Ardia *et al.*, 2011; Knutie *et al.*, 2017b; Knutie, 2020). In some situations, however, the pattern is more complicated. The structure of immune defences of roe deer (*Capreolus capreolus*) changes with physiological condition but whether an immune defence increases or decreases differs among defences (Gilot-Fromont *et al.*, 2012), although nutritional condition as defined herein was not measured. Similarly, antibody concentrations in Soay sheep (*Ovis aries*) are positively, negatively and non-linearly associated with body mass depending on the antibody type measured (Nussey *et al.*, 2014). North American elk (*Cervus elaphus*) from a high-density population and adult elk have lower nutritional condition and higher constitutive, antibacterial defences than those from low-density populations and yearling elk, respectively (Downs *et al.*, 2015). Thus, individuals adjust their investment in immune strategies, depending on their nutritional condition, but the pattern of investment is situational. Understanding when and how this switch occurs is critical for predicting the reproductive number, R_0_, for the spread of a parasite through a population and the long-term persistence of both the host and the parasite.

Interactions among nutritional condition, immune defences and parasites are complex. The energy and nutrients ingested by a host supports both the host’s immune system and reproduction of parasites. Cressler *et al*. (2014) modelled resource allocation between the host’s immune system and parasites. When resources were allocated to the host’s immune system first, parasite load peaked at low acquisition rates and then declined with increasing acquisition rates. When parasites were prioritized, parasite load increased with increasing energy acquisition, and when hosts and parasites competed over resources parasite load peaked at intermediate resource acquisition (Cressler *et al.*, 2014). Evidence for all three models exists in the empirical literature (Cressler *et al.*, 2014), and differences in patterns of allocation to hosts and parasites will complicate management and intervention plans for species of concern. As examples from species of low conservation concern, laboratory mice (C57BL/6) maintained outside in enclosures and experimentally infected with a gastrointestinal nematode (*Trichuris muris*) had reduced rates of weight gain per time spent foraging, suggesting either acquisition of additional food might be prioritized to parasites or greater energy expenditure to fight the infection (Budischak *et al.*, 2018). Additionally, female red deer (*C. elaphus*) did not reduce investment in immunity during lactation, but did have increased parasite intensity (Albery *et al.*, 2020), suggesting parasites might have increased access to resources during lactation. Finally, mallards in better nutritional condition shed more influenza virus (Arsnoe *et al.*, 2011). For all of these examples, resource supplementation might lead to increased host competence and parasite spread. Thus, understanding how resources are partitioned between hosts and parasites becomes critical for developing management plans for species of concern. When food availability and parasites are suspected to be driving population declines, screening for parasite infections (see Beechler *et al.*, 2019 for an example of screening) and quantifying immune responses to changes in interventions will allow managers to develop better plans.

### Ecoimmunology and disease ecology in bats

Chiroptera (bats) is the second largest mammalian group after rodents, with more than 1400 species described to date (Wilson and Mittermeier, 2019). They occupy almost every habitat on all continents except Antarctica, show an incredibly diverse ecology and provide humans with crucial ecosystem services, such as fruit pollination, seed dispersal (Ghanem and Voigt, 2012) and pest control (Maine and Boyles, 2015). Recently, bats became the focus for human-oriented approaches in infectious disease research as important reservoirs for various emerging viruses.

Bats host the most zoonotic viruses, including Ebola, rabies, Nipah and Hendra viruses (Olival *et al.*, 2017) and SARS-related coronaviruses (Jo *et al.*, 2020), and in most cases the infection causes little to no pathology. The lack of clinical symptoms might be associated with tolerance or resistance towards intracellular pathogens, including viruses (Brook and Dobson, 2015; Goh et al. 2020; Guito et al. 2021). Indeed, some bats constitutively express type I interferons with antiviral activities (Mandl *et al.*, 2018; Gorbunova *et al.*, 2020), but not all (Streicker and Gilbert, 2020). Moreover, to combat inflammation-associated pathologies, some bat species demonstrate reduced production of pro-inflammatory cytokines TNFα and IL-1β via various mechanisms (Gorbunova et al., 2020), which are associated with high anti-inflammatory cytokine IL-10 expression (Kacprzyk *et al.*, 2017).

Besides their role as reservoirs, bats are susceptible to extracellular pathogens such as bacteria and fungi (Mühldorfer, 2013; Brook and Dobson, 2015). One example is *Pseudogymnoascus destructans*, the cold-loving fungal pathogen causing the disease ‘white nose syndrome’. This disease has been responsible for mass mortalities in cave-dwelling hibernating North American bats with important conservation and economic consequences (Hecht-Höger *et al.*, 2020). *Pseudogymnoascus destructans* originates from Europe and recent studies indicate that North American bats exhibit a local and systemic immune response towards the fungus (Johnson *et al.*, 2015, Field *et al.*, 2015; Lilley *et al.*, 2019; Hecht-Höger *et al.*, 2020), while European bats have evolved tolerance mechanisms (Bandouchova *et al.*, 2018; Lilley *et al.*, 2019; Hecht-Höger *et al.*, 2020; Fritze *et al.*, 2021).

Bats share the same distribution and often food resources with humans, causing human–bat conflicts, which will probably increase in the future. The potential involvement of bats in the current COVID-19 pandemic and other disease outbreaks might lead to further persecutions and intentional killing of bats by humans (Fenton *et al.*, 2020). According to the International Union for the Conservation of Nature, habitat destruction, hunting, infectious disease and persecution threaten vulnerable bat populations (Fenton *et al.*, 2020). The impact of these anthropogenic factors on bat conservation differs in magnitude between species and is linked to their ecology. Foliage-roosting Bornean bat species are particularly sensitive to habitat destruction, showing reduced body condition and a decrease in immune cell numbers (Seltmann *et al.*, 2017), while man-made roosts negatively affect the adaptive immunity of *Tadarida brasiliensis* (Allen *et al.*, 2009). Changing resource availability (e.g. roosting sites, food sources) increases the shedding of Henipaviruses (e.g. Hendra and Nipah viruses) in fruit bats, which can not only increase viral transmission between bats, but can also lead to spillover into secondary and tertiary hosts (Plowright *et al*., 2008; reviewed in Kessler *et al*., 2018). Besides biological pollution (see above the case of *P. destructans*), bats are affected by chemical pollutants, which are already causing immunosuppression at low levels (Becker *et al.*, 2017). The epidemiological consequence of these negative effects is unknown.

Despite the methodological difficulties of working on bat immunology (Schountz, 2014), there has been significant progress in recent years, which will continue especially with the continuous development of novel species-non-specific assays and -omics technologies. A deeper knowledge of bat immune mechanisms and factors influencing their immunity is important not only to forecast reservoir species and prioritize surveillance targets (Becker *et al.*, 2020b), but also for conservation efforts to predict vulnerable species, to understand the effects of sub-lethal threatening factors and their epidemiological consequences.

**Figure 1:**
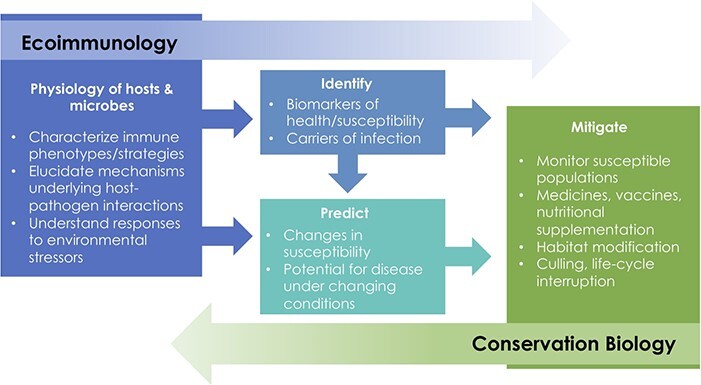
The conceptual link between ecoimmunology and conservation biology in understanding how hosts and pathogens will respond to anthropogenic change. Ecoimmunology involves the measurement of host physiology, including their microbes. This in turn allows us to ‘identify’ biomarkers of disease and potential carriers of infection and ‘predict’ what causes changes in susceptibility and leads to disease in wild populations. Finally, we can use these ecoimmunological tools to develop ‘mitigation’ strategies, which may involve, for example, population monitoring, vaccine development and/or nutritional/microbial supplementation, habitat modification and/or culling and life-cycle interruption.

## Future directions: filling the gaps for conservation success

Ecoimmunology offers conservation scientists and practitioners with the opportunity to expand our understanding of animal health to improve predictions and manage the impacts of anthropogenic environmental change on individuals, populations, species and communities. The utility of physiology for conservation biology lies in its ability to derive testable hypotheses by combining ecoimmunological approaches with traditional ecological metrics (population monitoring, biodiversity assessments and animal growth and abundance), and emerging or novel tools (next generation molecular tools, -omics, biotelemetry, citizen science), to understand the importance of mechanisms (Carey, 2005; Cooke and O'Connor, 2010; Cooke *et al.*, 2017; Madliger *et al.*, 2020). While the case studies presented in this paper speak to the diverse ways in which ecoimmunological tools and approaches are being used to inform and manage conservation efforts in a variety of taxa, there remain key gaps in our understanding of how immune functions respond to environmental change and how we can use ecoimmunological tools to better manage issues of conservation significance.

As a diverse group of ecoimmunologists with a range of expertise and an understanding of conservation issues that could benefit from ecoimmunological approaches, we have developed a series of research questions that we hope may guide future research to improve the utility of ecoimmunology for conservation and biodiversity protection. While not an exhaustive list, these questions span a range of topics from the fundamental impacts of anthropogenic changes on immune function, to the development of novel testing technologies, to understanding how inter-individual variation in immune responses shape population level responses to anthropogenic change. Moreover, these questions are deliberately broad, meaning that there are likely many sub-questions that can be developed from each of these overarching questions. To arrive at the 10 final questions, each author was independently invited to propose 3–5 research priority questions that they felt were important to direct future research in the field and to better integrate ecoimmunological tools into traditional conservation biology approaches (Figure 1). We collected 43 questions from the authorship and grouped these into broad themes (Supplement 1). We then condensed the questions into 10 focal questions that captured the intent of the original proposed questions. The final questions were then circulated to the authorship for comment and approval. They are presented below under the subheadings Identifying drivers of immune phenotypes, Predicting outcomes and Mitigation.

### identifying drivers of immune phenotypes

1. What roles do early-life stressors have on later-life health and disease susceptibility in wildlife?

2. How does anthropogenic driven environmental change [e.g. pollutants (noise, light, microplastics, chemical), land use changes, abiotic factors, environmental instability] affect the function and competency of the immune system? How predictable are responses to environmental stressors across individuals/populations/species/taxa?

3. How do we integrate ecoimmunology with other disciplines, such as animal behaviour, endocrinology, metabolism, genetics and species interactions (e.g. host-microbiome, community dynamics) to create a more holistic approach to conservation?

### Predicting outcomes

1. What role do immune strategies (avoidance, tolerance, resistance) have in determining disease risk? Can we predict ‘at-risk’ populations/species/taxa from their immune strategy?

2. How can advancing medical and analytical technologies (e.g. high-throughput molecular technologies, ‘omics’, health monitoring, etc.) improve the use/uptake of ecoimmunological tools in conservation?

3. How do we more effectively link anthropogenic-mediated changes in immune function to changes in disease susceptibility and other fitness consequences? What traits do we measure and how do we best measure them?

4. How do individual-level differences in immune defences affect disease processes within/between populations? How generalizable are patterns across populations?

5. What is the role of immunity in range expansion, biological invasions or adaptation to human-influenced environments (e.g. urban, agricultural areas)?

### Mitigation

1. How can we improve immunocompetence in threatened species (e.g. vaccine development, immune ‘boosting’ agents)? Can we leverage investment in human vaccines to improve interventions/outcomes for threatened species?

2. What ecoimmunological tools can be used in environmental remediation approaches to address the realized and potential disease problems caused by anthropogenic change?

The immune system, like other physiological systems, is exquisitely sensitive to the environment and as an organismal-level process (Esser, 2016), can help us monitor and manage the impacts of environmental change in near real time (Acevedo-Whitehouse and Duffus, 2009). Unlike traditional ecological monitoring approaches, the effects of environmental change on immune systems can be swift (Hill-Cawthorne and Sorrell, 2016) and can potentially be detected before effects manifest at the population or ecosystem levels. Indeed, the diversity of immunological responses to environmental change means that ecoimmunologists are well positioned to contribute to the prediction, management and recovery of biodiversity loss resulting from anthropogenic environmental change. However, a key issue for the development of ecoimmunology as a conservation tool remains: what traits do we measure? The dynamism of the immune system, combined with its extensive interconnectivity with other physiological and morphological systems and the high degree of inter-individual variability, means that any immune parameter measured in isolation likely has little value (French *et al.*, 2009; Heinrich *et al.*, 2017).

Individuals, populations and species can have vastly different immune response strategies and pathogen exposure histories (Becker *et al.*, 2020a), immune strategies may also change across life history stages (Lee, 2006; Rollins-Smith, 2017; Love *et al.*, 2008) and host–pathogen relationships may vary both spatially and temporally (Boëte *et al.*, 2019, Altizer *et al.*, 2018), which in turn can influence resulting immune responses. Moreover, the utility of some traditional ecoimmunological metrics for predicting the outcome of a pathogen exposure is largely unknown (Hawley and Altizer, 2011). Indeed, how metrics that are measured at the level of the individual ‘scale-up’ to provide a picture of population- or species-level disease responses, remains a critical gap in our understanding. Increasingly, ecoimmunologists are taking a multivariate approach that not only considers different aspects of immune function, but also captures the inter-individual, spatial and temporal variations in immune system traits (Brock *et al.*, 2013; Neuman-Lee *et al.*, 2019; Becker *et al.*, 2020a). Moreover, integrating ecoimmunological metrics with physiological, behavioural and microbiome studies could identify underlying relationships that may serve as indices of immune function (Campbell *et al.*, 2018; Neuman-Lee *et al.*, 2019). This integration might be particularly useful for rare/cryptic, threatened or small animals for which traditional ecoimmunological tools (e.g. blood sampling) are considered too invasive or impractical. Equally, they may reveal trade-offs with immune function that may affect the host’s capacity to respond to infections (Demas *et al.*, 2012; Brace *et al.*, 2017). Emerging technologies that allow rapid, cost-effective sequencing of immune gene expression and advances in wearable ‘health monitoring’ devices will allow unprecedented access to the immune systems of free-ranging animals over both temporal and biological scales. Currently, ecoimmunological data are heavily biassed towards birds and certain mammals (e.g. rodents, ungulates), and comparative data for other vertebrates and invertebrates is relatively scarce (Cooke *et al.*, 2020). Studying the diversity of immune strategies should lead to better models for conserving animals facing disease challenges.

The scale and rate of current environmental changes means that many conventional wildlife management approaches are likely to be unsuitable for managing future environmental remediation and rewilding interventions (Cooke *et al.*, 2020). Although ecoimmunology is not routinely considered as a wildlife management tool alongside traditional population and ecological assessment approaches, it has much to offer the field beyond simply monitoring pathogen and parasite loads. For example, threatened species at risk of disease-associated declines due to the loss of genetic diversity may benefit from nutritional supplements (including supplemental feeding, provision of key vitamins and minerals, antioxidants, bioflavonoids) and vaccination programs (Strandin *et al.*, 2018; Newton *et al.*, 2019). Likewise, health screening that includes metrics of immunological fitness could improve the outcome of translocation and reintroduction programs and reduce the likelihood of disease emergence and spread (Ballou, 1993; Teixeira *et al.*, 2007; Tarszisz *et al.*, 2014). Investment in wildlife health programs has the added benefit of potentially reducing disease spread to humans and domestic animals. In the face of the recent SARS-COV-2 virus outbreak, a ‘One-Health’ approach that incorporates ecoimmunological approaches to monitor immune parameters and pathogen loads in wildlife at the growing human–wildlife interface will allow us to better manage the emergence of novel zoonoses (Watsa, 2020; Zinsstag *et al.*, 2020). As the rate of emerging diseases continues to grow in both the human population and in wildlife (Jones *et al.*, 2008), ecoimmunological tools and approaches will be critical for predicting and managing disease risks and pathogen spread.

To conclude, ecoimmunology offers a range of opportunities to describe the effects of anthropogenic change on wildlife, to better manage its impacts on ecosystems and to identify solutions to the problems (Madliger *et al.*, 2016). In the case studies provided herein, we have demonstrated the diverse ways that ecoimmunological tools are currently addressing pressing conservation concerns. We have also identified key avenues for future research that will improve how we can use ecoimmunological knowledge to better manage issues of conservation significance. As with conservation physiology more broadly, the value of ecoimmunology as a tool to remedy the biodiversity crisis will depend largely on effective collaboration and communication with other conservation practitioners, policy makers and the broader community. Mixed or conflicting messaging can create a discord between conservation requirements and public health recommendations. For example, tick habitat and Lyme disease transmission can be reduced via cut grass lawns (e.g. Centers for Disease Control and Prevention, https://www.cdc.gov/lyme/prev/in_the_yard.html), but these lawns are ecologically depauperate habitats for biodiversity (Smith *et al.*, 2015). Ecoimmunology can play an important role to both identify these discords, and to help inform and create policy that effectively meets the needs of both.

## Funding

This work was supported by an Australian Research Council Discovery Grant (DP190102152) to C.E.F and R.L.C.

## Supplementary Material

Supplement_1_Original_Questions_coab074
