# Supplementary material for: Applied ecoimmunology: using immunological tools to improve conservation efforts in a changing world
Source: Conserv Physiol. 2021 Sep 7;9(1):coab074. doi: 10.1093/conphys/coab074 (PMC8422949; doi:10.1093/conphys/coab074)
Supplement: Supplement_1_Original_Questions_coab074 [file supplement_1_original_questions_coab074.docx]

**Supplement 1. Original submitted questions**

1. How do early life history stressors influence later life immune function?
2. What role do epigenetic modifications have for immune function and disease susceptibility in wildlife?
3. How is immunological variation linked to land-use changes, chemical pollution, etc.; i.e. what are the most important spatial drivers of immunity?
4. What role does exposure to low-levels of environmental toxins play in disease dynamics? What are the population-level consequences of these synergistic effects for persistence of species of concern.
5. How do emerging/novel/increasingly significant pollutants (e.g. noise, light, microplastics, hormones) affect the performance of immune system functions?
6. What are the immunological consequences of various anthropogenic factors (land use changes, chemical and physical pollution) on various species? How generalizable are these?
7. How do multi-stressor interactions and effects (synergistic, antagonistic etc) influence immune function?
8. How does the stability/instability of the changing environment affect immunity?
9. How do we develop better measures of the temperature dependence of immune function, to better predict the effects of climate on disease dynamics?
10. How well do markers of immune fitness translate between species? What kind of predictive power do they have?
11. What role do environmentally-driven shifts between tolerance and resistance strategies play in outbreaks of established infectious diseases?
12. Are species that use certain immune strategies more likely to become species of conservation concern?
13. What are the limits of plasticity of the immune system in dealing with a changing environment?
14. Is boosting the immune system of captive animals the best approach to improve health or are there side-effects (e.g. oxidative damage, interaction with reproductive hormones) that might reduce effectiveness of conservation action?
15. How can we improve immunocompetency in threatened species e.g. vaccine development for at risk species /threatened species and conservation, including ensuring animals in captive breeding and release programs are immunocompetent.
16. Can we bolster animal immune defences against novel or significant pathogens I.e. Using ecoimmunology to develop “vaccinations” against parasites of concern?
17. Can immunology and vaccines be used to control wildlife disease?
18. Can vaccines and therapeutics for emerging infectious diseases be developed faster through investigations of reservoir species immunology?
19. Can minimally invasive high throughput molecular technologies such as RNAseq or metabolomics be used to improve rapid pathogen surveillance, identify ‘at-risk’ individuals or populations or detect pathogen ‘super-spreaders’?
20. Will ‘omics’ technologies’ allow eco-immunologists to bridge the gap with mainstream immunology by lowering the reagent barrier?
21. Immunity is often studied in isolation from other important physiological functions. How do we better integrate ecoimmunology with other established disciplines like ecological endocrinology or the ecology of antioxidants and of oxidative stress?
22. How can behavioural observations supplement/work together with traditional eco immunological monitoring to describe and monitor ‘stress’ non-invasively in target organisms?
23. Can real-world immunology improve biomedical research translation?
24. Is there a trade-off between immune function and animal performance (locomotor performance/development and growth)?
25. What is the energetic cost of immune function?
26. How do environmental drivers influence the microbiome which in turn influences immune function and disease susceptibility?
27. How does the microbiome influence parasite/pathogen susceptibility? Does answering this question provide an opportunity for management interventions? What would those interventions look like?
28. What is the predictive power of single immune markers in terms of individual (or population) fitness consequences? or is it rather better to combine multiple markers into a single metric? If yes, which is the best approach to mathematically combine info from multiple markers?
29. How well do our measurements of immunity in the lab translate to the field? This may be especially important in animals that are difficult to collect/sample in the field
30. How do we more effectively link changes in immune function associated with anthropogenic activities to changes in disease susceptibility; what traits to measure and how do we best measure them?
31. How the measured immune markers predict fitness consequences, especially survival? Are there unique markers or marker combinations (genetic, physiological and immunological) which can advise on resilience of a species? Can these makers be used for conservation programs, e.g. predict success of translocations or releases into the wild?
32. How realistic are relatively short-term, non-invasive stress markers like corticosteroid levels, for managing long-term immune function/disease susceptibility?
33. Methodological development needs to continue, with special focus on species non-specific assays and use of non-invasive samples. Most of the current information is obtained from plasma and serum samples (e.g. due to storage possibilities on the field) however cellular immunity is largely neglected.
34. With the development of techniques using non-invasive samples, especially for endangered species, we need to understand the differences between local and systemic immunity, on how to interpret results from non-invasive samples.
35. Can ecoimmunology be used to assist in surveillance for emerging zoonoses, or better yet for predicting risks in host and vectors / modelling zoonotic emergence?
36. What ecoimmunological tools can be used to design/assess/manage environmental remediation approaches that directly address the realised and potential disease problems caused by habitat loss/fragmentation?
37. Can ecoimmunological tools be used to design effective and highly specific controls for invasive species management?
38. In a given species, does reduction in immunological variation due to population size contraction favour release, spread, and spillover of pathogens?
39. Most studies focus on single populations, use cross-sectional study designs; longitudinal approaches associated with large-scale/multi population studies would make findings more generalizable.
40. How do individual differences in immune defences affect fitness, and how do these differences scale up to influence population dynamics? That is, how do individual-level differences in immune defences affect population persistence?
41. What is the relative role of individual-level vs. ecological processes in determining disease dynamics? Does the relative effect of these processes differ for populations/species that are or are not susceptible to pathogen/parasite?
42. What is the role of immunity in range expansion, biological invasions, or adaptiveness to urban habitat?
43. What is the role of immunity in range expansion/ invasion and urbanization of certain wildlife species?
